# Protein synthesis and degradation are required for the incorporation of modified information into the pre-existing object-location memory

**DOI:** 10.1186/1756-6606-3-1

**Published:** 2010-01-08

**Authors:** Jun-Hyeok Choi, Jung-Eun Kim, Bong-Kiun Kaang

**Affiliations:** 1 National Creative Research Initiative Center for Memory, Department of Biological Sciences, College of Natural Sciences, Seoul National University, 151-742 Seoul, Korea; 2 Department of Brain and Cognitive Sciences, College of Natural Sciences, Seoul National University, 151-742 Seoul, Korea

## Abstract

Although some reports indicate that protein synthesis dependent process may be induced by updating information, the role of protein synthesis and degradation in changing the content of pre-existing memory is yet unclear. In this study, we utilized an object rearrangement task, in which partial information related to a pre-existing memory is changed, promoting memory modification. Inhibitors of both protein synthesis and protein degradation impaired adequate incorporation of the altered information, each in a distinctive way. These results indicate that protein synthesis and degradation play key roles in memory modification.

## Introduction

Memory formation in real life usually involves partial change of pre-existing memory. Though numerous studies have highlighted the molecular mechanism of memory formation, it is largely unknown how previously formed memory is altered or updated in the process.

A candidate mechanism of memory updating could involve dynamic regulation of memory stability after memory reactivation. Conventionally, it was thought that memory is consolidated by a protein synthesis-dependent process only once per item and persists thereafter in a stable state. However, accumulating evidence suggests that memory reactivation induces a reconsolidating process that depends on the protein synthesis [1-4]. While protein synthesis has been shown to be required for the memory restabilization after retrieval, the destabilizing phase seems to require protein degradation. These studies showed that, using the contextual fear memory task, protein degradation in the hippocampus after memory reactivation is required for weakening or strengthening of the fear memory [5,6]. This dynamic protein turnover after memory reactivation is hypothesized to be a molecular mechanism through which memory is updated or modified. Indeed, several reports have suggested that this protein-synthesis-inhibitor-sensitive reconsolidation process follows after reactivation only when there is an additional external stimulus that promotes updating of the original information [7-10].

However, the previous studies only focused on the strength of the memory for demonstrating the requirement of protein synthesis and degradation in the incorporation of changed information. Another major memory updating process in real-life is partially modifying the content of the initial memory rather than simply strengthening or weakening the memory. In the present study, we aimed to reveal the role of protein synthesis and degradation in the incorporation of partially modified information into the pre-existing memory, by using an object rearrangement task.

## Results

**Figure 1:**
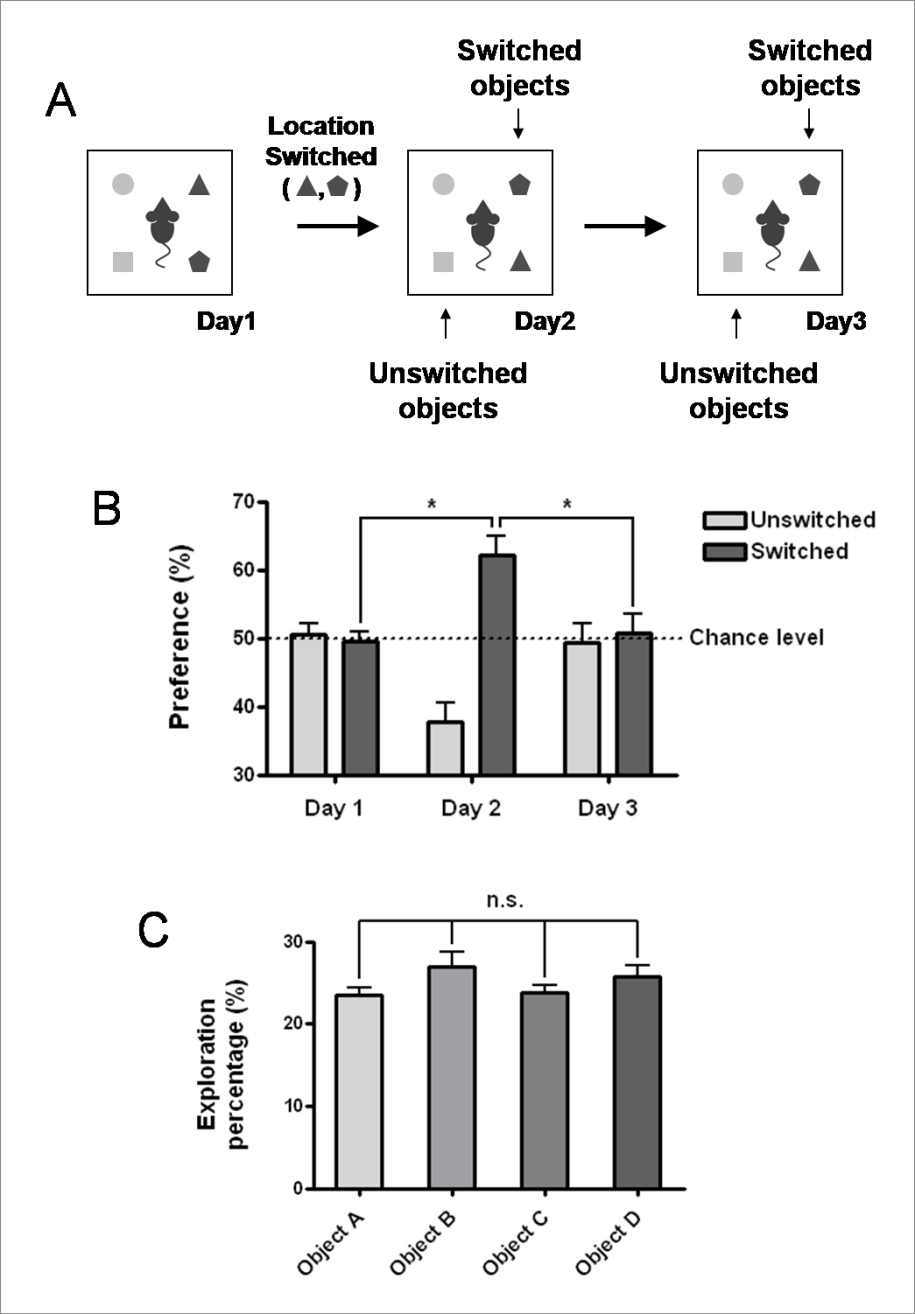
**Object rearrangement task**. **A**, Schematic view of the task. After five days of habituation to the context, the mice were exposed to four distinctive objects each placed in one of the four positions for 15 minutes (Day 1). Two adjacent objects' positions were switched for the next two days (Day 2, Day 3), exposed for 15 minutes each. The object pair that is switched was counterbalanced between experiments. **B**, Preference to unswitched and switched objects over 3 days (n = 6, *p < 0.05; paired t test). Preference is the percentage of the two switched/unswitched objects exploration time from the total exploration time. **C**, Preference to each objects in the first day (n.s., non significant).

To assess the incorporation of changed information into the preexisting memory, we chose an object-location memory task [11]. This task measures object-location associative memory utilizing the innate tendency of rodents to explore the novel aspects of the environment. The scheme of the task is depicted in Figure 1A. Nine- to 11-week-old C57BL/6N male mice were used for all the experiments, housed as described previously [12]. After five days of 15 minutes habituation to a context, the mouse was exposed for 15 minutes to four objects located in one of the four positions in the context. Twenty-four hours later, the mouse was re-exposed to the context for 15 minutes with the location of two adjacent objects inter-switched while the other two left unchanged. Higher explorative preference to the switched objects was expected due to the novelty of the situation. On the next day, the mouse was re-exposed for 15 minutes to the same object configuration as the second day. If it had successfully incorporated the changed information on the second day, there would be no novel aspects in each object anymore, resulting in similar preference for each of them.

To evaluate the preference for switched/unswitched objects, we measured the duration of exploration time for each object and calculated the percentage of the two switched/unswitched objects exploration time from the total exploration time. The preference to the switched objects was higher on day 2, indicating that the subjects have well recognized the original position or configuration of each object (Figure 1B). This returned to chance level at day 3 when the same configuration as day 2 was given. This implies that the subjects have well memorized the new, changed configuration to recognize it as familiar.

Although there was a slight difference in the preference for each of the four objects at the first day, it was statistically non-significant (Figure 1C). The two objects with slightly higher preference were always paired with the objects with lower preference, and the pair itself was counterbalanced for the next experiment.

**Figure 2:**
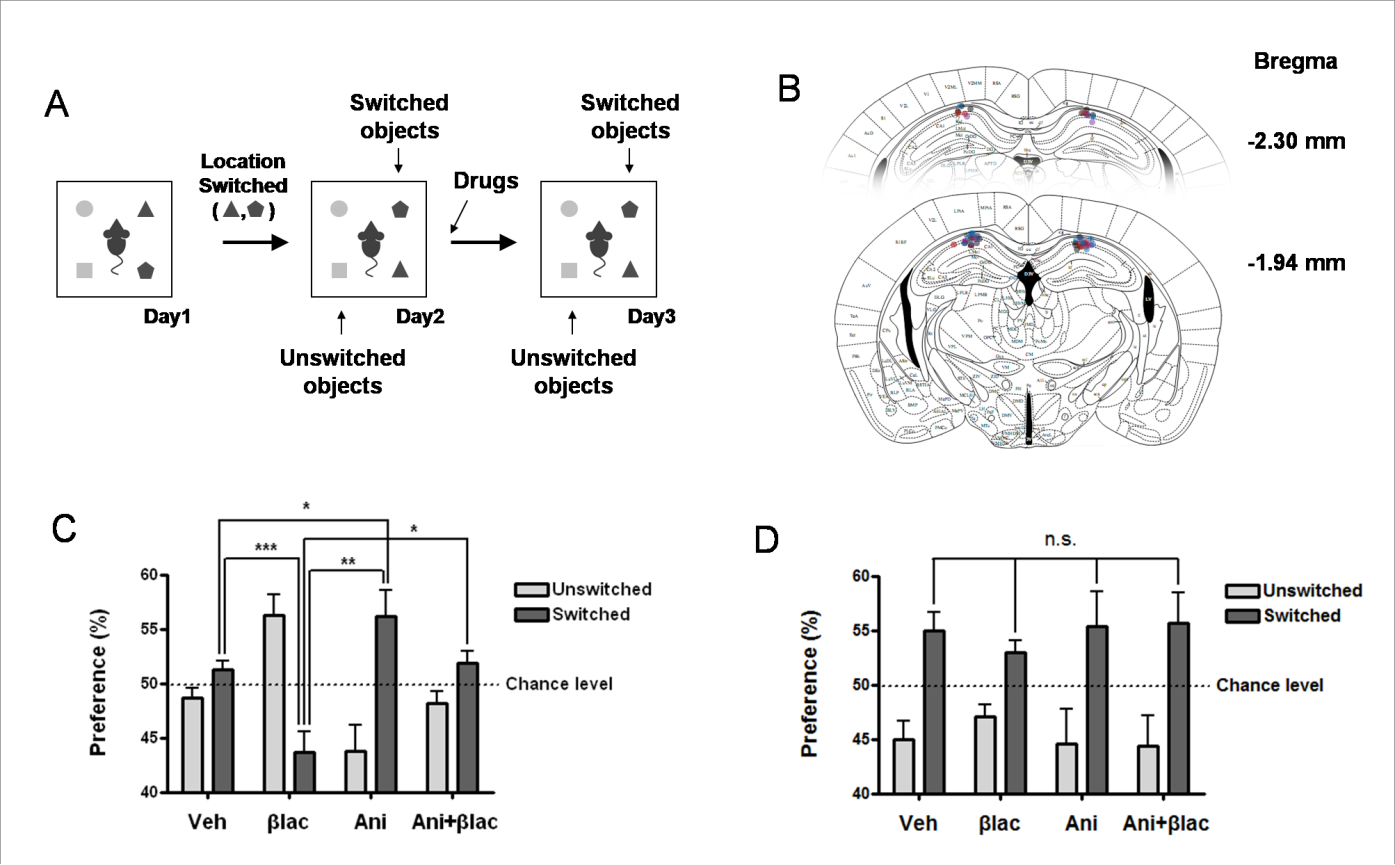
**Protein synthesis and degradation is required to appropriately incorporate partially modified information**. **A**, Schematic view of the task. The process is similar as in Figure 1A, except that the mice have received intrahippocampal injection right after day 2 exposure. **B**, Cannula location in the hippocampus, at two different rostro-caudal planes. Numbers indicate the posterior direction from the bregma. Grey, blue, red, and purple circles indicate the infusion site of vehicle, βlac, anisomycin, and βlac plus anisomycin double infusion group, respectively. **C**, Preference to unswitched and switched objects at the third day (Veh, vehicle, n = 10; Ani, anisomycin, n = 7; βlac, clasto-lactacystin-β-lactone, n = 7; Ani+ βlac, n = 4. F = 9.869, p = 0.0002; one-way ANOVA. *p < 0.05, **p < 0.01, ***p < 0.001; unpaired t test). **D**, Preference to unswitched and switched objects at the second day.

As our aim was to focus on the associative memory between the objects and their location, we targeted hippocampus which seems to be more specifically involved in object-location memory [13,14]. Hippocampus is known to be important for object-location memory [14-17]; although other regions such as mPFC and perirhinal cortex are also implicated in such tasks, they are probably more involved in novelty processing. Especially, it was recently reported that PKMzeta inhibitor destructed object location memory, but not object identity memory, when it was locally applied in hippocampus [18]. We implanted guide cannulae one week before the behavioral procedure and injected the protein synthesis inhibitor anisomycin (Ani; 200 μg/μl in aCSF, 0.3 μl) and/or the proteasome inhibitor clasto-lactacystin-β-lactone (βlac; 32 ng/μl in aCSF, 0.3 μl) or vehicle (Veh; aCSF, 0.3 μl) in the CA1 region of dorsal hippocampus, bilaterally, right after the exposure to the changed object location on day 2 (Figure 2A). Position of the cannulae tips are shown in Figure 2B.

On day 3, the anisomycin-injected group showed higher preference to the switched object compared to the vehicle-injected control group (Figure 2C), suggesting that subjects could not incorporate the information from the modified object configuration on day 2. On the other hand, the βlac-injected group showed higher preference to the unswitched object compared to vehicle control group. This suggests that the preexisting information (unchanged configuration) was relatively weakened or somewhat impaired compared to the new information (changed configuration). This is consistent with the findings that βlac interrupted the reconsolidation process (Lee et al, 2008). It is still possible that the new information was more effectively stored in the presence of βlac, thus increasing the relative familiarity with the switched objects over with the unswitched objects. It is noteworthy that the effects of anisomycin and βlac on the memory change were opposite [preference to the switched objects (anisomycin) vs. to the unswitched (βlac)] as are the opposite effects of these drugs on protein level]. When anisomycin and βlac were injected together, the preference was similar to the control group. The treatment of anisomycin and βlac at the same time offset their effects on the behavioral change. The preference for switched object, on day 2 was similar among the four groups (Figure 2D). Taken together, our data suggest that protein synthesis and degradation have important roles in regulating the process of modifying the previously formed memory.

## Discussion

In this study, we have provided a novel scheme of object-in-place memory task to assess whether the mice have correctly changed the contents of pre-existing object-location associative memory, making it possible to investigate the process of modifying memory. In previous studies focusing on the modification of pre-formed memory, the memory task commonly involved strengthening the memory by repeated training over days or weakening it by an experimental extinction protocol. Our modified object rearrangement task has an advantage that the memory is not simply altered in its strength, but that the partial contents of the pre-formed memory are rapidly reorganized to incorporate changed information. Based on this advantage, this task is likely to be valuable for studies on dynamic memory modification process. Using this task, we have found that protein synthesis and degradation are required for incorporating partially modified information into the pre-existing memory.

Considering the effect of anisomycin on the memory modification, the original memory might be expected to be destabilized by anisomycin after reactivation on day 2 as it would undergo a protein synthesis dependent reconsolidation process, possibly resulting in the chance level preference for each object on day 3. However, anisomycin-injected mice acted on day 3 as if the second object configuration is novel, resulting in higher preference for the switched objects. One possible explanation for this is that not every component of the related memory becomes labile in this stage to be sensitive to protein synthesis inhibitor. For example, the memories for unswitched objects might have been less affected by anisomycin being maintained relatively intact, while the memories for switched objects may have been more affected, being reactivated and destabilized. This may result in higher explorative preference for the switched objects, as the location for switched objects becomes more novel than the location for the unswitched objects. It is also possible that the consolidation-like process for incorporating additional information (i.e. switched location) is more sensitive to protein synthesis inhibitor than the reconsolidation process. In any case, our result indicates that incorporation of changed information into the pre-existing memory requires *de novo *protein synthesis.

The interpretation of the effect of proteasome inhibition is more complicated. If the process to incorporate the changed information is a simple additional learning of the novel information, the effect of a certain drug might be expected to be either impairment of modification leaving the memory unchanged or no effect leading to successful incorporation of the new information. In this study, however, the inhibition of protein degradation appeared to affect even the memory for unchanged information. Therefore, memory reactivation on day 2 is likely to have induced complex processes involving modification to the previous memory, in which the protein degradation plays a major role.

Previous reports suggest that destabilization of the reactivated memory precedes the protein synthesis-dependent process. However, the retrieval process of four-object rearrangement task differs from that of contextual fear conditioning as it incorporates additional information rather than simply recovers the memory state. Although the memory reactivation-induced protein degradation seems to be critical to form successfully modified memory, the full process may also, in parts, include consolidation-like process to encode the novel memory components. This process may be induced independently of destabilization of the previous memory. In this case, protein degradation inhibition would impair destabilization of the reactivated memory and possibly the following restabilization process, without affecting the independent consolidation-like process. The imbalance between these two essential components induced by protein degradation inhibition might have lead to higher preference for the unswitched objects on day 3. In addition, the protein degradation might have a unique role in either restabilization of the previous memory or in a consolidation-like process to incorporate additional information. We cannot exclude the possibility that protein degradation is involved in the consolidation process in the four-object task used in the present study, although it does not seems to be the case, at least acutely, in contextual fear memory [5].

We have locally treated the inhibitors in hippocampus based on references that indicate hippocampus as a region specifically involved in object location memory rather than the object identity memory. The results using PKMzeta inhibitor demonstrates that hippocampus is at least one of the memory storage sites for object location memory while it is not a critical storage site for object identity memory [18]. Therefore, protein synthesis and degradation inhibition treatment in hippocampus is unlikely to have affected the object identity memory. However, there is a possibility that other brain regions are also critically involved in this memory task.

Although more studies are required to fully understand the mechanism, the present study suggests that protein synthesis and degradation play important roles in modifying the memory in object-location memory paradigm. This evidence supports the hypothesis that memory dynamically changes after retrieval through protein turnover. In addition, we expect that the scheme used here or other modes of the object rearrangement task can be utilized for the investigation into the memory modification mechanism in the future.

## Competing interests

The authors declare that they have no competing interests.

## Authors' contributions

JHC: experiment design and implementation, manuscript writing.

JEK: behavior experiments and sample preparation

BKK: experiment design and advice, manuscript writing.

All authors read and approved the final manuscript.

## References

[B1] Suzuki A Josselyn SA Frankland PW Masushige S Silva AJ Kida S Memory reconsolidation and extinction have distinct temporal and biochemical signatures J Neurosci 2004 24 4787 4795 10.1523/JNEUROSCI.5491-03.2004 15152039 PMC6729467

[B2] Nader K Schafe GE Le Doux JE Fear memories require protein synthesis in the amygdala for reconsolidation after retrieval Nature 2000 406 722 726 10.1038/35021052 10963596

[B3] Milekic MH Alberini CM Temporally graded requirement for protein synthesis following memory reactivation Neuron 2002 36 521 525 10.1016/S0896-6273(02)00976-5 12408853

[B4] Dudai Y Reconsolidation: the advantage of being refocused Curr Opin Neurobiol 2006 16 174 178 10.1016/j.conb.2006.03.010 16563730

[B5] Lee SH Choi JH Lee N Lee HR Kim JI Yu NK Choi SL Kim H Kaang BK Synaptic protein degradation underlies destabilization of retrieved fear memory Science 2008 319 1253 1256 10.1126/science.1150541 18258863

[B6] Helton TD Otsuka T Lee MC Mu Y Ehlers MD Pruning and loss of excitatory synapses by the parkin ubiquitin ligase Proc Natl Acad Sci USA 2008 105 19492 19497 10.1073/pnas.0802280105 19033459 PMC2614788

[B7] Rodriguez-Ortiz CJ De la Cruz V Gutierrez R Bermudez-Rattoni F Protein synthesis underlies post-retrieval memory consolidation to a restricted degree only when updated information is obtained Learn Mem 2005 12 533 537 10.1101/lm.94505 16166395 PMC1240066

[B8] Morris RG Inglis J Ainge JA Olverman HJ Tulloch J Dudai Y Kelly PA Memory reconsolidation: sensitivity of spatial memory to inhibition of protein synthesis in dorsal hippocampus during encoding and retrieval Neuron 2006 50 479 489 10.1016/j.neuron.2006.04.012 16675401

[B9] Rossato JI Bevilaqua LR Myskiw JC Medina JH Izquierdo I Cammarota M On the role of hippocampal protein synthesis in the consolidation and reconsolidation of object recognition memory Learn Mem 2007 14 36 46 10.1101/lm.422607 17272651 PMC1838544

[B10] Rodriguez-Ortiz CJ Garcia-DeLaTorre P Benavidez E Ballesteros MA Bermudez-Rattoni F Intrahippocampal anisomycin infusions disrupt previously consolidated spatial memory only when memory is updated Neurobiol Learn Mem 2008 89 352 359 10.1016/j.nlm.2007.10.004 18054256

[B11] Dix SL Aggleton JP Extending the spontaneous preference test of recognition: evidence of object-location and object-context recognition Behav Brain Res 1999 99 191 200 10.1016/S0166-4328(98)00079-5 10512585

[B12] Ko HG Jang DJ Son J Kwak C Choi JH Ji YH Lee YS Son H Kaang BK Effect of ablated hippocampal neurogenesis on the formation and extinction of contextual fear memory Mol Brain 2009 2 1 10.1186/1756-6606-2-1 19138433 PMC2629467

[B13] Jenkins TA Amin E Pearce JM Brown MW Aggleton JP Novel spatial arrangements of familiar visual stimuli promote activity in the rat hippocampal formation but not the parahippocampal cortices: a c-fos expression study Neuroscience 2004 124 43 52 10.1016/j.neuroscience.2003.11.024 14960338

[B14] Bachevalier J Nemanic S Memory for spatial location and object-place associations are differently processed by the hippocampal formation, parahippocampal areas TH/TF and perirhinal cortex Hippocampus 2008 18 64 80 10.1002/hipo.20369 17924520

[B15] Lee I Solivan F The roles of the medial prefrontal cortex and hippocampus in a spatial paired-association task Learn Mem 2008 15 357 367 10.1101/lm.902708 18463175 PMC2364607

[B16] Gilbert PE Kesner RP Memory for objects and their locations: the role of the hippocampus in retention of object-place associations Neurobiol Learn Mem 2004 81 39 45 10.1016/S1074-7427(03)00069-8 14670357

[B17] Gilbert PE Kesner RP Role of the rodent hippocampus in paired-associate learning involving associations between a stimulus and a spatial location Behav Neurosci 2002 116 63 71 10.1037/0735-7044.116.1.63 11895184

[B18] Hardt O Migues PV Hastings M Wong J Nader K PKMzeta maintains 1-day- and 6-day-old long-term object location but not object identity memory in dorsal hippocampus Hippocampus 2009 in press 19806657 10.1002/hipo.20708

